# Adrenocortical Adenocarcinoma in a Syrian Golden Hamster (*Mesocricetus auratus)* With Suspected Hyperadrenocorticism

**DOI:** 10.1002/vms3.70895

**Published:** 2026-04-13

**Authors:** Mathilde Firmin Billaux, Sabrina Vieu, Antoine Lecomte, Marie Cuvelier, Marine Rohel, Jérôme Abadie, Nicolas Soetart

**Affiliations:** ^1^ Oniris, CHUV Nantes France; ^2^ Oniris, INRAE, BIOEPAR Nantes France; ^3^ Oniris, LabOniris Nantes France

**Keywords:** adenocarcinoma, adrenal tumour, hamster, hyperadrenocorticism, neuroendocrine

## Abstract

Although adrenal gland tumours are among the most common neoplasms in hamsters, few cases have been reported. A 2‐year‐old intact female pet golden hamster (*Mesocricetus auratus*) was initially presented with marked non‐pruritic alopecia of the hindquarters. Physical examination and ultrasonography revealed a large, unilateral, right‐sided abdominal mass measuring 2 cm in diameter. A complete blood count, serum biochemistry, urinary cortisol‐to‐creatinine ratio (UCCR) and fine‐needle aspiration of the mass were subsequently performed. Cytological examination revealed an epithelial tumour with endocrine differentiation, and both the biochemical profile and UCCR suggested the possibility of a corticosteroid‐secreting tumour. Due to the location and invasive nature of the mass, complete excision was not feasible. Postmortem histopathology confirmed an adrenocortical adenocarcinoma with chronic, marked, diffuse atrophic dermatosis. Antemortem diagnosis was achieved using laboratory testing and diagnostic imaging, although such procedures are rarely performed in this species.

## Graphical abstract

A 2‐year‐old female golden hamster presented with non‐pruritic alopecia of the hindquarters. Diagnostic imaging and cytological evaluation revealed a unilateral adrenal mass consistent with an adrenocortical tumour, while biochemical analyses suggested corticosteroid secretion. Postmortem histopathological examination confirmed an adrenocortical adenocarcinoma associated with diffuse atrophic dermatosis. These findings demonstrate the value of a combined diagnostic approach, integrating clinical examination, imaging, cytology and hormonal assays, for identifying adrenal tumours in pet hamsters.

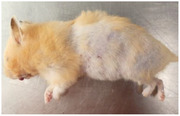

## 1 Case Report

A 2‐year‐old intact female pet golden hamster (*Mesocricetus auratus*) was presented to the veterinary teaching hospital for non‐pruritic truncal abdominal alopecia and suspected polydipsia progressing over 4 months. Adequate environmental conditions and an appropriate diet were reported. The hamster was in good body condition and weighed 140 g. External physical examination revealed bilateral, symmetrical alopecia of the flanks, abdomen, hindquarters and tail, with no other associated lesions. A firm, painless, ovoid mass measuring approximately 1–2 cm in diameter was palpated in the right abdomen. The rest of the clinical examination was unremarkable.

Based on these findings, abdominal ultrasonography (L15‐7io compact linear array transducer, PHILIPS Affiniti 70G, 7‐15 MHz) was performed under gas anaesthesia (2.5% isoflurane in 100% oxygen at 1 L/min). A peritoneal mass measuring 1.7 cm consistent with the right adrenal gland or the right ovary, a mild diffuse hepatomegaly with multiple sub‐centimetric cysts in various lobes, and a moderate amount of anechoic peritoneal effusion were observed.

**FIGURE 1:**
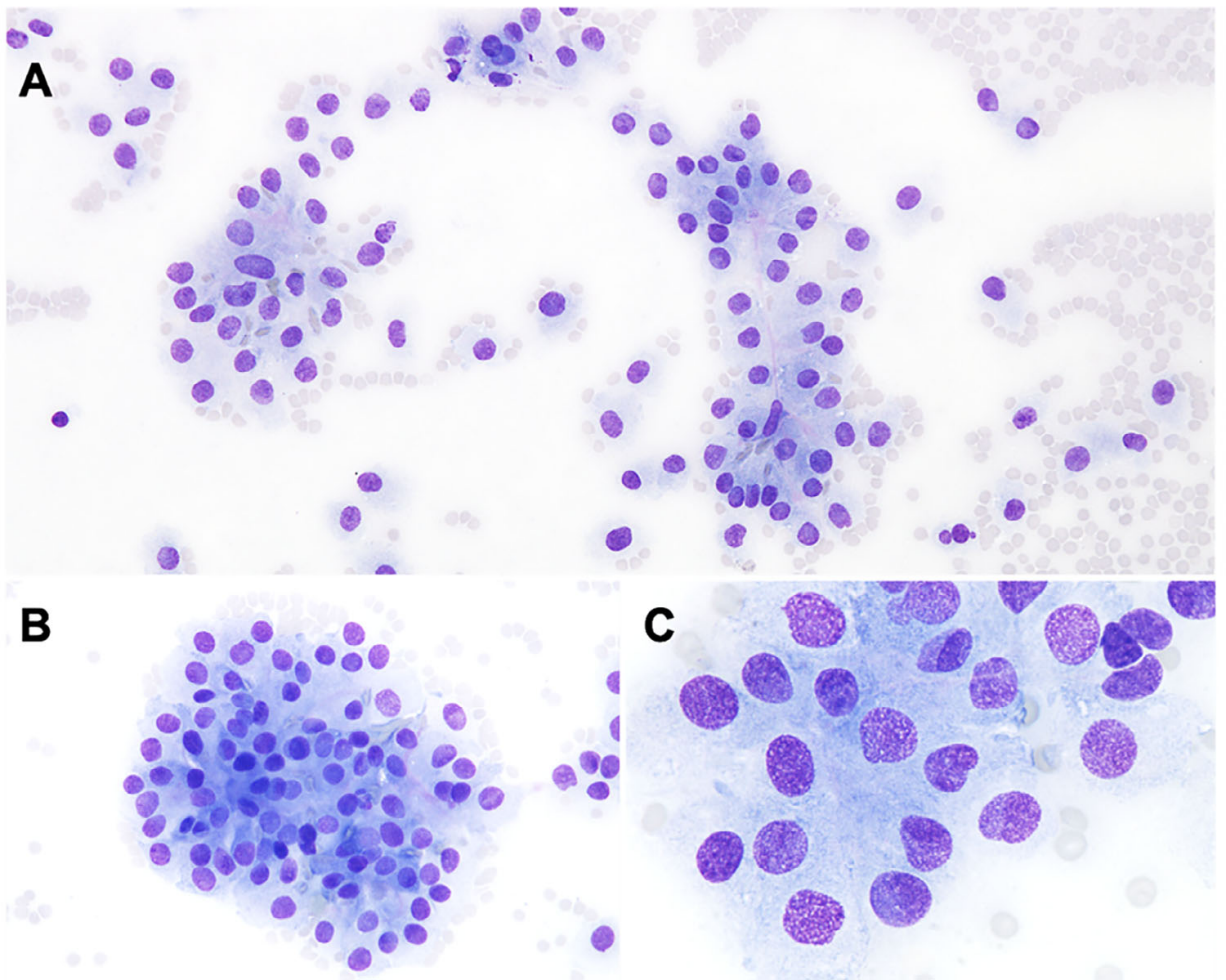
Photomicrographs of the abdominal mass fine needle aspiration smear. May‐Grünwald Giemsa. (A) ×20 objective, (B) ×40 objective, (C) ×100 objective. (A, B) Loosely cohesive clusters of cells with rosette‐like arrangement, rarely containing a very small amount of magenta amorphous material. (C) Oval to polygonal cells with indistinct borders (endocrine appearance).

A fine‐needle aspiration cytology of the abdominal mass was performed. Slides were stained with May‐Grünwald‐Giemsa (MGG) stain. Samples were highly cellular with mild haemodilution and a moderate number of free nuclei. Numerous loosely cohesive clusters of nucleated cells, frequently forming rosette‐like arrangements around rare, very small amounts of magenta amorphous material, were observed. These cells were oval to polygonal with variably defined (mostly indistinct) borders, and a moderate nuclear‐to‐cytoplasmic ratio. Nuclei were round to ovoid with finely stippled chromatin, containing occasional variably sized nucleoli. The cytoplasm was moderately abundant, lightly basophilic and homogeneous. Cells displayed moderate anisocytosis and anisokaryosis, with few mitotic figures and rare binucleated cells (Figure 1). Cytological interpretation was consistent with an epithelial neoplasm with endocrine appearance. Due to its localisation, the most likely hypotheses were an adrenocortical or, less probably, an ovarian tumour.

Complete blood count, plasma biochemistry and electrolyte panel (Table 1) showed mild anaemia with slight leukopenia, increased alkaline phosphatase activity, hyperuraemia and hypokalaemia, with no other biochemical abnormalities, according to published reference values (Carpenter and Harms 2022). To explore the hypothesis of hyperadrenocorticism, resting blood cortisol concentration was measured (Cortisol RIA, Demeditec, radioimmunoassay): a value of 18 nmol/L, within the reference interval, was measured. Urinary cortisol‐to‐creatinine ratio (UCCR), a screening test widely used in dogs to rule out or confirm Cushing's syndrome (Behrend et al. 2013, Nagata et al. 2022), was 56.6 × 10^−6^ (urinary cortisol: 73 nmol/L; urinary creatinine: 1.29 mmol/L). As no species‐specific threshold exists for this test, the value was compared to the threshold established in dogs, where a value < 20 × 10^−6^ rules out hypercortisolism. In this case, UCCR was thus considered increased.

**TABLE 1:** Hematology and biochemistry for a hamster with adrenal tumour.

Blood parameter	Units	Results	Reference intervals (Carpenter and Harms 2022, Lindstrom et al. 2015) (min–max)
Total protein	g/L	61	56–62
Albumin	g/L	29	38–44
Aspartate Aminotransferase (AST)	U/L	48	30–76
Alanine Aminotransferase (ALT)	U/L	46	34.7–71.3
Alkaline phosphatase (ALP)	U/L	256*	120–132
Creatinine	µmol/L	26.52	26.52–61.88
Urea	mmol/L	1.66*	8.2–11.8
Triglycerides	mmol/L	1.43	1.749–2.915
Cholesterol	mmol/L	2.86	3.198–5.07
Cortisol	nmol/L	17.94	13.8–27.6
Sodium	mmol/L	144	144–152
Potassium	mmol/L	3.2*	5.7–7.1
Chloride	mmol/L	100	96–104
Hematocrit	%	37*	45–50
Hemoglobin	g/dL	12.7*	16.6–18.6
Red Blood cells	10^6^/µL	6.4*	7–8
White blood cells	10^3^ cells/µL	5*	7–10
Neutrophils	10^3^/µL %	1.7 34	N/A 18–40
Lymphocytes	10^3^/µL %	2.4 49*	N/A 56–80
Monocytes	10^3^/µL %	0.8 16	N/A 2
Eosinophils	10^3^/µL %	<0.1 1	N/A 0–1
Basophils	10^3^/µL %	<0.1 <1	N/A 0–1
Platelets	10^3^/µL	569	N/A
UCCR	10^−6^	56.6	N/A

*Note*: Asterisk means out of reference range.

A deterioration in the hamster's general condition was noticed by the owner 1 week later. Considering the poor prognosis, the animal was euthanised with an injection of 1 mL of pentobarbital into the gingival vein, and a necropsy was performed with the owner's consent. Organs were systematically sampled and fixed in 10% neutral buffered formalin for histological examination.

**FIGURE 2:**
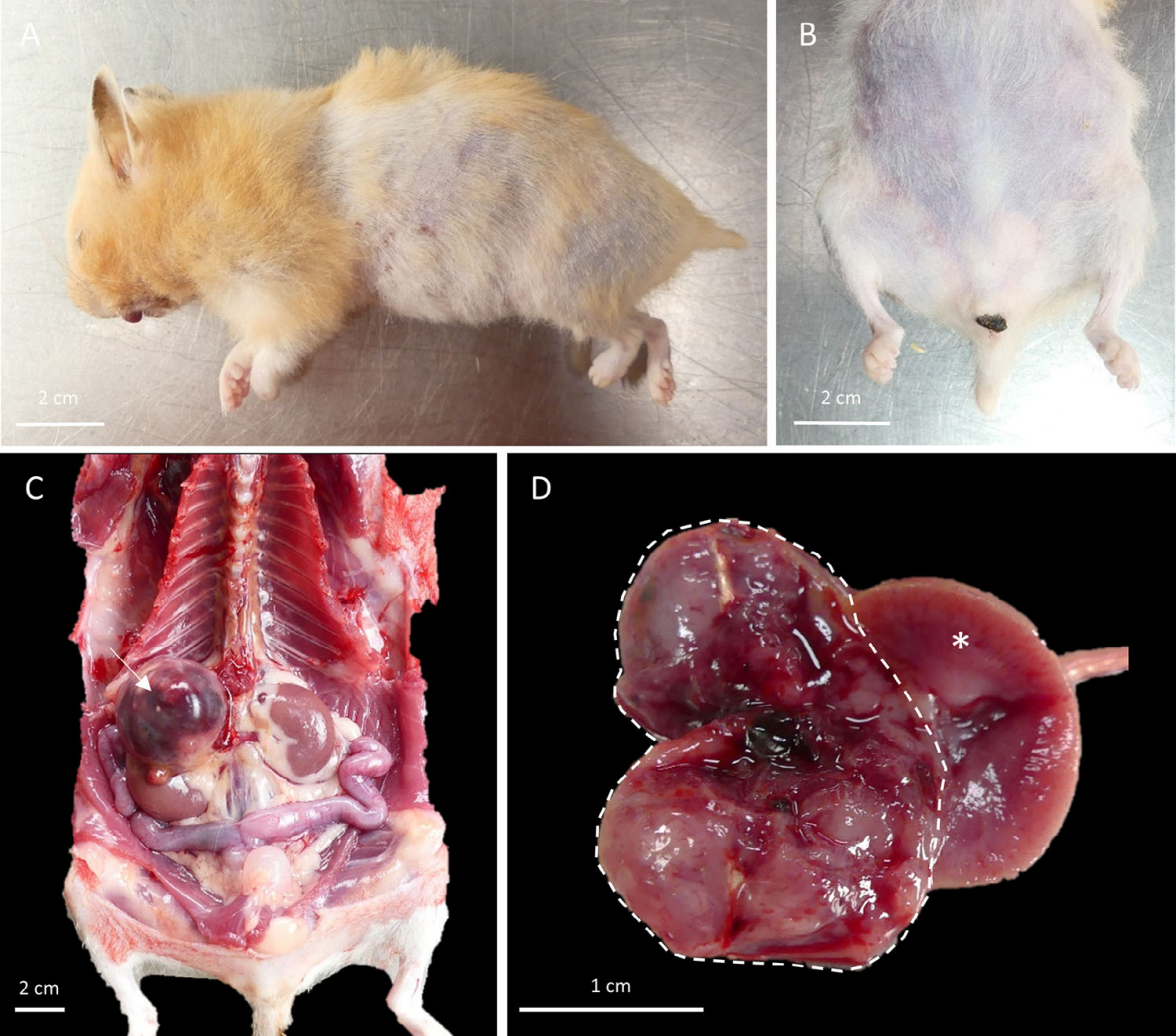
Photographs of the necropsy of a golden hamster with an adrenal mass and hyperadrenocorticism. (A, B) Non‐pruritic alopecia of the flanks, abdomen, pelvic limbs, and tail. (C) A nodular mass of 1.5 x 2 x 2 cm located in the anatomic area of the right adrenal gland (arrow). (D) On cut section, the mass was nodular (dashed line) with central necrotic and haemorrhagic remodelling and compression of the right kidney (asterisk).

At necropsy, the dermatological lesions (truncular alopecia) were observed as previously described (Figure 2A,B). A gingival haematoma was present, compatible with the pentobarbital injection site. The abdominal cavity showed a small amount of clear, non‐viscous, slightly blood‐tinged fluid. A nodular mass of approximately 1.5 × 2 × 2 cm was located in the anatomical region of the right adrenal gland and infiltrated the cranial pole of the adjacent kidney. Tumour growth with marked central necrosis and haemorrhage severely remodelled the mass with macroscopic effacement of adrenal cortico‐medullary architecture (Figure 2C,D). Biliary cysts were observed in liver parenchyma. The pituitary gland was macroscopically unremarkable.

**FIGURE 3:**
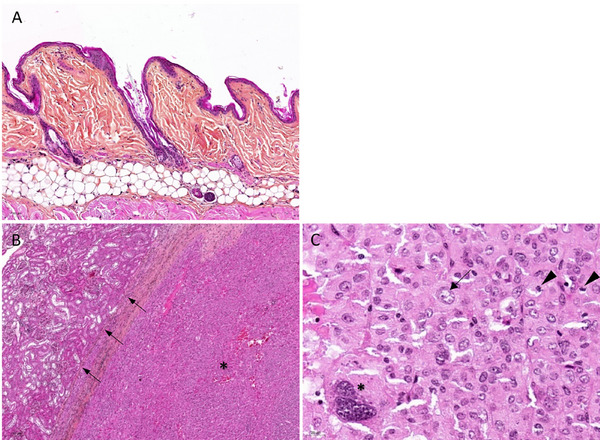
Photomicrographs of histologic sections of skin and adrenal gland mass in a golden hamster with hyperadrenocorticism. Hematoxylin and eosin with safran. (A) Marked atrophy of the epidermis and pilosebaceous appendages, with moderate orthokeratotic hyperkeratosis. Hair follicles are predominantly in the telogen phase. (B, C) The adrenal mass was encapsulated, with pressure atrophy on the adjacent renal parenchyma (arrows). It was composed of a dense population of polygonal cells arranged in sheets and cords, in a well‐vascularised stroma (asterisk). (C) Cellular atypia was marked, with anisocytosis, anisokaryosis, karyomegaly (arrow), nuclear hypochromatism, prominent nucleolation and numerous multinucleated cells with 2 or 3 nuclei (asterisk). The mitotic index was moderate, about 2 mitoses per field ×400 (0.237 mm^2^) (arrowheads).

On histological examination, the adrenal gland showed a well‐defined, encapsulated, multinodular to coalescing neoplastic lesion, composed of a dense population of polygonal cells arranged in sheets and cords, in a scant but highly vascularised stroma with frequent vascular ectasia. Pressure atrophy was present in the adjacent renal parenchyma (Figure 3B). Neoplastic cells averaged 20–30 µm in size, with ill‐defined margins, abundant eosinophilic and homogeneous cytoplasm, a regular 8 to 12 µm ovoid central nucleus with fine dusty to vesicular chromatin, and a discrete nucleolus. Cellular atypia was marked, with anisocytosis, anisokaryosis, karyomegaly, nuclear hypochromatism, prominent nucleoli and numerous multinucleated cells with 2 to 3 nuclei. The mitotic index was moderate, about 2 mitoses per high‐power field (×400, 0.237 mm^2^) (Figure 3C). Mild lymphoplasmacytic infiltrates were multifocally distributed within the tumour. Acute to chronic necrotic and haemorrhagic changes were present multifocally in the tumour, in association with the formation of fibrous tissue containing numerous hypersegmented neutrophilic granulocytes, lymphocytes, epithelioid macrophages and haemosiderophages. A diffuse and marked atrophy of the left adrenal gland cortex was noted.

**FIGURE 4:**
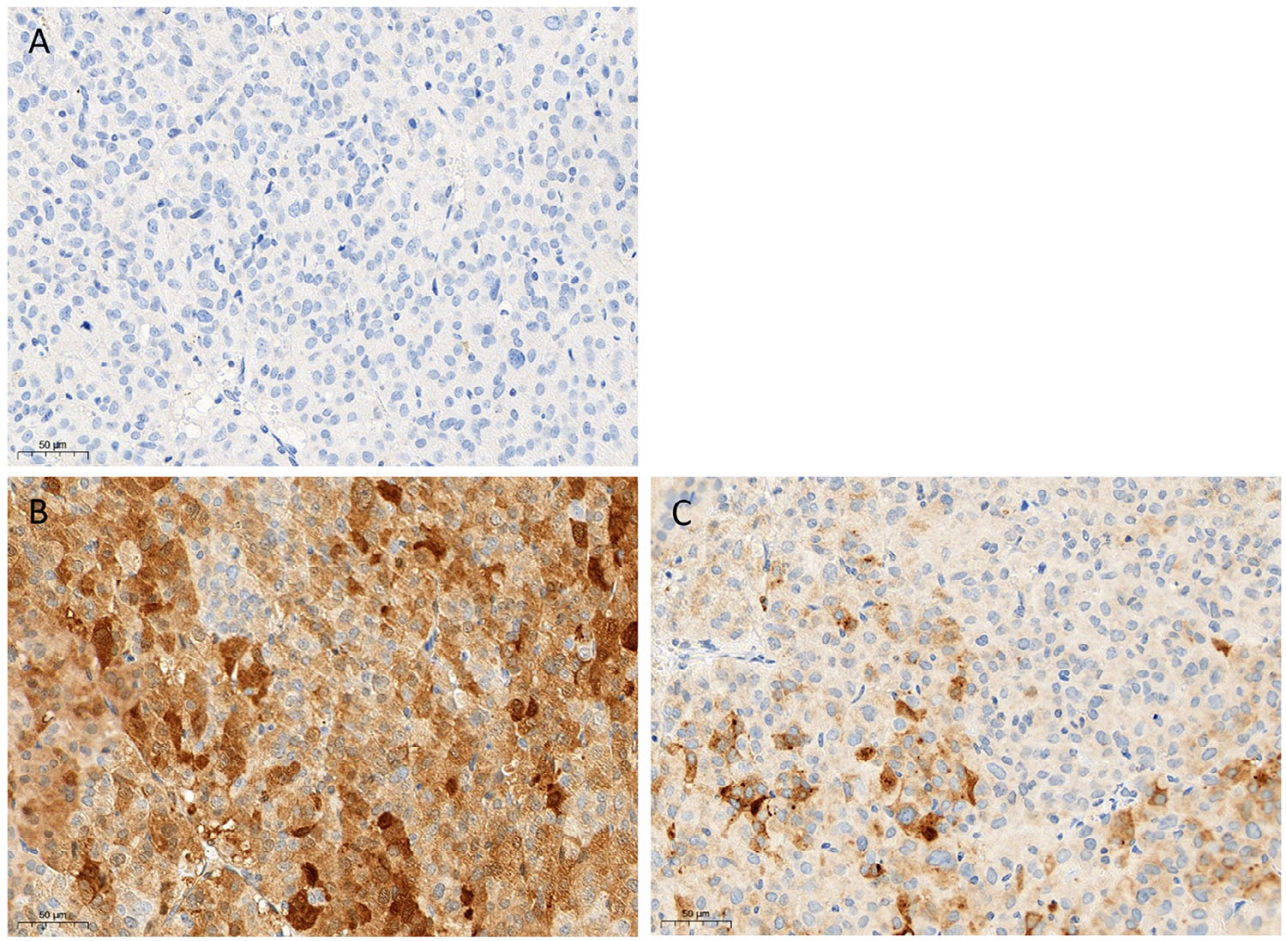
Photomicrographs of neoplastic cells immunohistochemical profile in the adrenal mass. (A) Neoplastic cells showed no expression for Pancytokeratin (AE1/AE3), (B) a moderate to strong cytoplasmic expression for neuron specific enolase, (C) and a variable mild to strong cytoplasmic expression for synaptophysin, compatible with an endocrine tumour developed from the adrenal cortex.

Immunohistochemistry was used to investigate the histogenesis of neoplastic cells. No expression of pancytokeratin (epithelial cell marker; murine monoclonal antibody, clones AE1 and AE3, Dako M3515) was observed, and a constant mild to strong cytoplasmic labelling for Neuron‐Specific Enolase (NSE, neuroendocrine cell marker; murine monoclonal antibody, clone BBS/NC/VI‐H14, Dako) was detected, as well as a cytoplasmic labelling for synaptophysin (neuroendocrine cell marker; rabbit polyclonal antibody, Bio SB, BSB5948) of variable intensity, ranging from mild to moderate in half of the neoplastic cells (Figure 4A–C). These characteristics are consistent with an endocrine neoplasm arising from the adrenal cortex. Regarding the skin, a diffuse moderate orthokeratotic hyperkeratosis of the epidermal *stratum corneum* was observed extending to the hair follicles (infundibular keratin plugs or comedones) and the spinous and granular layers were reduced to about two to four cell layers, with marked atrophy of the pilosebaceous appendages. Hair follicles were extensively and predominantly in the telogen phase, with or without visible hair shafts (Figure 3A). Some hair follicles contained irregular sections of adults and eggs of metazoan parasitic arthropods compatible with *Demodex criceti*.

## 2 Discussion

Based on clinical signs and pathological findings, this hamster was diagnosed with an adrenocortical adenocarcinoma presenting with clinical and biological features consistent with hyperadrenocorticism. According to a 2016 study on disorders and mortality of 16,605 hamsters in the United Kingdom, including 73.45% Syrian hamsters (*M. auratus*), abdominal tumours and masses were major causes of mortality in the Syrian hamster, accounting for 5.71% and 7.08% of deaths, respectively (O'Neill et al. 2022), A small number of retrospective studies are available reporting the incidence of neoplasia in hamsters. The most common neoplasms reported in pet hamsters were haematopoietic tumours and tumours involving the integumentary system (Kondo et al. 2008, Rother et al. 2021). Although few case reports are available, detailed clinical descriptions and antemortem diagnostic investigations of adrenal tumours remain scarce. Their recognition is clinically relevant, as endocrine dysfunction can lead to non‐specific signs such as alopecia or weight loss. Documenting such cases contributes to a better understanding of their biological behaviour and diagnostic features and may ultimately improve clinical management and treatment strategies. This case report, therefore, aims to describe and discuss the presentation of an adrenal tumour in this species, providing clinical, biological, imaging, cytological, and histopathological details to enrich the existing literature.

Hyperadrenocorticism develops secondary to a pituitary or adrenocortical secretory tumour, or following iatrogenic exposure to corticosteroids. Corticosteroid synthesis is regulated by the corticotropic system (hypothalamus, adenohypophysis, and adrenal zona fasciculata). The adrenal cortex of hamsters secretes both cortisol and corticosterone, with their relative proportions exhibiting significant diurnal variation. Moreover, glucocorticoid secretion in hamsters occurs at a much lower rate compared with other mammals (Bielinska et al. 2009).

Tumours of the adrenal gland have been reported in a wide variety of animals, including dogs (Jekl 2025), cats, ferrets, goats (Collins 2008), as well as in myomorph rodents such as hamsters, rats and gerbils (Kamino et al. 2001). In Syrian hamsters, adrenal gland tumours predominantly affect the cortex, as observed in the present case, while medullary tumours are rare (Collins 2008).

The incidence of adrenocortical tumours (cortical adenoma or adenocarcinoma) in Syrian hamsters varies between studies from 0 in pet hamsters (Kondo et al. 2008, Rother et al. 2021) to more than 50% in laboratory hamsters (Collins 2008, Kamino et al. 2001, McInnes et al. 2013). In a 2001 study on laboratory animals, 66% of males and 38% of females were found to have adrenal adenomas or carcinomas (Kamino et al. 2001). Another study on this species reported an incidence of 14.5% for neoplastic lesions of the adrenal gland (Collins 2008). A study on 500 Syrian hamsters (control animals from carcinogenicity studies) showed that adrenal tumours were frequent, with incidences of 56.4% and 46% for adenomas, and 22% and 13.2% for carcinomas, in males and females, respectively (McInnes et al. 2013).

Previous studies and case reports on adrenal gland tumours in Syrian hamsters are mainly focused on postmortem characteristics with limited descriptions of clinical and biological consequences (Collins 2008, Greenacre 2004, Kamino et al. 2001, Kondo et al. 2008, McInnes et al. 2013, Orr and Lawrence 1984, Petrou 2022, Rother et al. 2021). A case series of hyperadrenocorticism described three hamsters presented with alopecia and hyperpigmentation. In one case, necropsy revealed an adrenocortical carcinoma; however, no antemortem diagnostic tests had been performed. The two remaining cases displayed elevated alkaline phosphatase activity, and one hamster also exhibited increased cortisol levels (the other one was not tested). Necropsy in that individual revealed a pituitary adenoma associated with bilateral adrenal cortex hyperplasia (Bauck et al. 1984). In our case, clinicopathological findings were also consistent with hyperadrenocorticism. In particular, concurrent hypokalaemia and low urea were most likely secondary to renal losses associated with long‐standing polyuria and polydipsia, which had been evolving over a 4‐month period and were suspected to be driven by hyperadrenocorticism. The CBC, showing mild anaemia and lymphopenia, was consistent with the haematologic patterns commonly described in hyperadrenocorticism and chronic stress responses across species. An increase in ALP activity has been previously reported in hamsters with hyperadrenocorticism (Bauck et al. 1984). The causes of this finding can be multiple. In healthy individuals, bone and intestinal isoenzymes are reported to be the most prevalent (Cox and Gökcen 1973). In our case, no bone or intestinal lesions were noted, so a hepatic origin was suspected, possibly due to corticosteroid‐induced cholestasis. A corticosteroid‐induced ALP isoenzyme, as described in dogs, has not been reported in hamsters; however, this aspect has never been specifically investigated and therefore cannot be completely ruled out.

In our case, alopecia was the only clinical sign detected, associated with diffuse atrophic dermatosis. Alopecia is a nonspecific finding but could be classically associated with endocrine disorders, including hyperadrenocorticism, in association with cutaneous atrophy, as already reported in hamsters (Bauck et al. 1984). However, the clinical manifestation of demodectic mange also includes alopecia. Demodex sp. infestation is usually facilitated by intercurrent conditions, particularly nutritional imbalance and immunosuppression, which can be a consequence of hyperadrenocorticism. In our case, both cutaneous atrophy and alopecia could be associated with hyperadrenocorticism, but it cannot be excluded that demodicosis infestation was promoted by cancer‐related or corticosteroid‐associated immune alterations and has at least participated in hair loss.

In our case, the cytologic examination of the mass revealed features consistent with an endocrine tumour. In many species, adrenocortical tumours display the classic ‘neuroendocrine pattern’ characterised by frequent naked nuclei in a background of abundant free cytoplasm, rosettes or pseudoacinar arrangements and cells containing numerous clear small to medium‐sized cytoplasmic lipid vacuoles (Bertazzolo 2020). However, a histological study reports a near absence of lipid vacuoles in the adrenal cortex of golden hamsters in contrast with other species, which aligns with our histological and cytological findings (Alpert 1950). The near absence of lipid vacuoles in the adrenocortical cells of hamsters may be, at least in part, attributed to their species‐specific cholesterol metabolism, resulting in adrenal glands containing very little stored cortical hormones or precursors (Lehoux and Lefebvre 1980).

No immunohistochemical investigations have been performed in previously published cases of adrenal cortical carcinomas in hamsters. Regarding the immunohistochemical profile of the neoplastic cells in the present case, the characteristics observed (i.e., no expression of pancytokeratin and moderate to strong NSE and synaptophysin expression by the tumour cells) are consistent with those described in cortical adrenal carcinomas in other species (DeLellis and Shin 2003, Lam 2021, Peterson II et al. 2003, Shigematsu et al. 2009). Notably, although synaptophysin is not expressed in non‐tumoural adrenal cortex, neoplastic transformation is associated with the expression of neuroendocrine markers, including Synaptophysin and NSE in humans and in ferrets. These immunohistochemical findings, supported by the clinical data, were therefore considered compatible with an adrenal cortical origin of the neoplasm in our case.

In humans, some common mutated genes are known to be associated with cortical adrenal carcinoma tumourigenesis, such as IGF‐2 or TP53 (Lam 2021). Such genetic alterations have not been extensively studied in naturally occurring cases in domestic animals. Consequently, we did not perform further molecular or genetic investigations in the current case.

A moderate and multifocal lymphocytic and plasmacytic infiltrate was observed within the neoplastic stroma. It is likely a part of the tumour immune microenvironment. In humans, it is thought to be less abundant in cortical adrenal carcinomas than in other tumours, and not associated with an efficient anti‐tumour immunity, possibly due to anti‐inflammatory effects of glucocorticoids produced by neoplastic cells (Landwehr et al. 2020). However, as these cells were not further characterised in our case, we cannot determine whether this population contributed to anti‐tumour immunity or promoted immune escape through tolerogenic signalling.

Finally, biliary cysts are lesions commonly found in ageing Syrian hamster livers (Cooper et al. 2021). They are thought to be related to a developmental anomaly that progresses with age and leads to biliary duct dilation. In our case, these cysts were not associated with major hepatic remodelling or clinical hepatic dysfunction and were therefore considered as an incidental finding.

The biological diagnosis of hyperadrenocorticism is challenging, as no test has a 100% diagnostic accuracy; it is based on either increased cortisol production and/or decreased sensitivity to the corticotropic system (hypothalamus, pituitary gland, adrenal gland) (Behrend et al. 2013).

In veterinary medicine, biological exploration of glucocorticoid‐secreting adrenocortical tumours often requires dynamic tests (e.g., low‐dose dexamethasone suppression test, ACTH stimulation test), which have not been described in hamsters. Alternatively, urinary cortisol:creatinine ratio (UCCR), has been reported in one Syrian hamster with suspected hyperadrenocorticism: a value of 45.6 × 10^−6^ was considered compatible with hyperadrenocorticism by the authors (Martinho 2006). The animal died without undergoing necropsy, and the existence of an adrenal or pituitary tumour could not be confirmed. In our case, biological analysis showed a low, considered normal, resting blood cortisol (18 nmol/L) and a UCCR value of 56.6 x10^−6^, interpreted as increased, but the lack of reference values in this species made a definitive diagnosis unattainable. Two hypotheses might explain the possible discrepancies between these two tests: (1) cortisol secretion may have been pulsatile and the blood sampling was collected in a non‐secreting phase: the resting basal cortisol value is indeed known to lack sensitivity and supports the usefulness of dynamic tests to diagnose hyperadrenocorticism; or (2) the adrenal tumour was secreting other steroid hormones with glucocorticoid activity, for example corticosterone (which is one of the main glucocorticoids secreted by hamster, along with cortisol), which is not detected by the radioimmunoassay blood test (data from the manufacturer indicate less than 5% cross‐reactivity of the antibodies with corticosterone) (Botía et al. 2023). One point to emphasise is the difficulty of carrying out complementary examinations, especially blood tests on hamsters, partly due to owner reluctance, despite their growing status as companion animals, and partly due to challenging venous access and the difficulty of obtaining sufficient blood volume to carry out blood tests (Case 2022, Martinho 2006). The volume of blood sampled must be large enough to perform the various blood tests required to support or exclude the different diagnostic hypotheses. In general, removal of a blood volume less than 10% of the body weight is safe (Miwa and Mayer 2021). Thus, non‐invasive methods, such as the measurement of urinary or faecal glucocorticoids, are of particular interest in small mammals, but the absence of validated methods and the lack of species‐specific reference intervals ​​make them difficult to interpret in the clinical setting.

This case highlights the clinical, cytological, and histopathological features of an adrenocortical adenocarcinoma in a pet Syrian hamster, associated with dermatological lesions suggestive of hyperadrenocorticism. While adrenal tumours are described in this species, antemortem diagnosis remains rare due to limited diagnostic tools and practical challenges. This report illustrates the potential value of combining imaging, cytology, and hormonal assays, despite the absence of species‐specific reference intervals, in achieving a presumptive diagnosis. Increasing awareness of endocrine disorders in small mammals may help improve diagnostic approaches and welfare in these increasingly popular companion animals.

## Author Contributions

M.F.B. and S.V. were responsible for case management. N.S., A.L., J.A., M.C. and M.R. carried out the anatomohistopathology diagnostic. The manuscript was written by M.F.B. and S.V. with contributions from the other authors. All the authors reviewed the manuscript.

## Funding

The authors have nothing to report.

## Ethics Statement

The authors have nothing to report.

## Conflicts of Interest

The authors declare no conflicts of interest.

## Data Availability

The data presented in this case report are available on request from the corresponding author.
